# Young green quinoa as a sustainable functional crop with anti-inflammatory effects in macrophage cells

**DOI:** 10.1038/s41598-025-03742-w

**Published:** 2025-06-26

**Authors:** Hamdan Khattib, Noam Pintel, Soliman Khatib, Lior Rubinovich, Dorit Avni

**Affiliations:** 1 Bio-Compounds and Immune-Mediated Diseases Group, MIGAL—Galilee Research Institute, 1101600 Kiryat Shemona, Israel; 2 Department of Biotechnology, Faculty of Science and Technology, Tel Hai Academic College, 1220800 Kiryat Shemona, Israel; 3 Northern Agriculture R&D, MIGAL - Galilee Research Institute, P.O. Box 831, Kiryat Shemona, Israel

**Keywords:** Inflammation, LPS, Immunology, Quinoa, IL-6/TNF-α, Active compounds, Sustainable crop, NCD, Cytokines, Resilient, Climate, Metabolomic, Biochemistry, Metabolomics, Immunology, Cytokines, Inflammation, Innate immune cells

## Abstract

Current staple crops such as rice, wheat, and maize dominate food systems but lack climate resilience, necessitating a shift toward nutrient-rich, sustainable alternatives. *Chenopodium quinoa*, (C. quinoa) has gained global recognition for its adaptability and nutritional value. However, while quinoa grains have been extensively studied, young green quinoa (YGQ) leaves remain underexplored despite their potential to enhance both agricultural sustainability and human health. This study investigates the anti-inflammatory properties of YGQ leaves extracts from eight quinoa accessions cultivated during the summer. Using lipopolysaccharide (LPS)-activated mouse macrophage cells (RAW264.7), we assessed the inhibition of key pro-inflammatory cytokines, tumor necrosis factor (TNF)-α and Interleukin (IL)-6. Four types of extracts—Ethanol:Water (70:30) (ETDW), Ethanol (ET), Ethyl Acetate (EA), and Hexane (HE) were prepared, revealing significant and specific IL-6 inhibition, with ETDW exhibiting the highest suppressive effect (73–100%). LC–MS/MS analysis identified flavonoids as the likely bioactive compounds responsible for this activity. Importantly, toxicity assays confirmed the extracts’ safety. These findings position YGQ leaves as a valuable natural source of bioactive compounds with potential applications in functional foods, which offer health benefits beyond basic nutrition by targeting the prevention of noncommunicable diseases and chronic inflammatory conditions. Furthermore, integrating YGQ leaves into food systems could support sustainable agriculture, as quinoa is a climate-resilient crop, providing dual benefits for public health and food security.

## Introduction

By the year 2050, according to the Food and Agriculture Organization (FAO), agriculture will face the challenge of providing the food and nutrition requirements of the world population, which is expected to reach 9 billion people and will require an increase of 70% of global food productionproduction^1^. This intensification will present several significant challenges, including mechanization and improved farming practices, maintaining productivity levels, adopting environmentally sustainable agriculture such as efficient water and land use, and minimizing negative environmental impacts of food production systems^2^.

Despite over 350,000 identified plant species^3^, only 255 plants significantly contribute to the human diet worldwide, with maize, wheat, and rice dominating global food production^1,4^ with 1194.80, 792.40, and 506.04 million tons per year, respectively^4^. However, these leading staple crops lack climate resilience and are linked to high-carbohydrate diets, which have been associated with metabolic disorders and non-communicable diseases (NCDs)^1^. As health-related research increases yearly, there is a growing interest in diversifying food sources with high-nutrient and climate-resilient crops. It is believed that in the upcoming decades, the human diet will undergo significant changes in crop yields and alternative crop preferences^5^. The search for alternative crops, such as quinoa, sorghum, millet, and amaranth, that may provide nutritional benefits while supporting sustainable agricultural practices is imperative^5,6^

Non-communicable diseases (NCDs) are the primary cause of death globally, accounting for 74% of all deaths each year, according to the World Health Organization^7^. NCDs are long-term conditions with slow progression caused by various genetic, behavioral, environmental, and physiological factors and are exacerbated by Western lifestyles and diets that have influenced the population significantly in the last few decades, contributing expressively to these diseases today^7,8^. Western diets such as fast food, snacks, processed food products, soft drinks, and on-the-go meals lack minerals, fiber, vitamins, and proteins. Underconsumption of critical nutrients leads to various diseases and chronic inflammations such as diabetes, non-alcoholic fatty liver disease, cardiovascular diseases, and cancer^9^. Addressing this requires a transformation in global dietary patterns and increase in the availability of healthy, functional foods for all socioeconomic levels^10^. Moreover, global challenges such as climate change, the COVID-19 pandemic, and geopolitical conflicts, emphasize the urgent need to secure sustainable and nutritious food sources^11^. One of the leading strategies that aims to deal with global challenges is the European Green Deal Farm to Fork Strategy. This unique effort takes an integrated approach to optimize the entire food supply chain, from crop production to consumer plates. It ensures food security, nutrition, and public health, making sure that everyone has access to sufficient, safe, nutritious, sustainable food^12^. It emphasizes reducing environmental impact and fostering healthier diets to curb NCD prevalence.

NCDs and chronic inflammation are mediated by key pro-inflammatory cytokines such as tumor necrosis factor (TNF)-α and Interleukin (IL)-6^13^ and have been proposed as a central player in inflammatory cell activation, recruitment, and development of many chronic inflammatory diseases^12^. As diet influences inflammation, identifying natural anti-inflammatory compounds for functional foods is an emerging research priority. Previous studies have discovered a range of metabolites from various plants that can be used to treat numerous targets and have considerable therapeutic efficacy or can potentially be prevented^14^.

Quinoa (*Chenopodium quinoa Wild.*), a resilient Andean crop cultivated for over 7000 years, presents a promising alternative^6,14^ Its great adaptability to harsh environments, tolerating frost, drought, and high soil salinity, make it a promising option for food security^6^. Additionally, quinoa contains various biologically active compounds, enhancing its versatility as a crop that not only contributes to sustainable agricultural practices but strengthens also food security^15,16^. Quinoa grains gained attention due to their high protein content, minerals, and vitamins, than other common crops. Quinoa provides a nutritious and affordable dietary option, particularly in regions where cultivating other nutrient-rich crops may be challenging. Because of its high genetic variety, quinoa can thrive in a wide range of agroecological conditions, and in recent years, it has been grown in many countries outside of the Andean region^16–18^.

Due to these phenomenal characteristics of quinoa, research has been accelerating in the fields of agriculture, novel food, food security, and environmental protection. Most quinoa-based research focuses on the grain nutrient composition, protein quality, and functional characteristics in quinoa-enriched food products. While quinoa grain research is extensive, its leaves remain underexplored despite their nutritional richness^15^.

Young green quinoa (YGQ) leaves are edible, protein-rich, and contain high levels of fiber, vitamins, and bioactive compounds^18–20^. In a recent study that took place in Israel, it was shown that YGQ can be grown in a desert-like area, with low irrigation, while maintaining high protein content. Interestingly, this study showed that the protein amount was higher than that of other crops, including quinoa grain^21^. These leaves contain bioactive compounds such as saponins, phytosterols, phytoecdysteroids, and phenols, which exhibit diverse biological activities, including anti-cancer, antioxidant, and anti-inflammatory properties^22^. Moreover, YGQ’s rapid growth cycle and high nutritional yield make it a potential solution for sustainable food production^15,23^. Assessing its above-mentioned potential, it could facilitate the geographic spread and expansion of young vegetative and step forward the Farm to Fork strategy.

This study investigates the biological activity of YGQ leaves from eight quinoa accessions to assess their potential to prevent inflammatory diseases. Using macrophage cells stimulated by lipopolysaccharide (LPS), a bacterial molecule linked to inflammation^24^, we evaluated the inhibition of TNF-α and IL-6 production and metabolomic profile. While previous studies have explored the health benefits of quinoa grains, research on quinoa greens remains limited, primarily focusing on their nutritional and phytochemical composition and potential health benefits. To our knowledge, this is the first study demonstrating the anti-inflammatory effects of YGQ leaves, identifying their bioactive compounds, and examining their impact on immune cell activity. Our findings indicate that the chemical components of YGQ leaves significantly influence activated macrophages and reduce pro-inflammatory cytokines, which may benefit human health^6^. Additionally, this study provides insights into the metabolomic profile of diverse YGQ accessions and highlights YGQ leaves as a valuable nutritional and medicinal resource, supporting further exploration of their applications in functional food development and human health^25^.

## Results

### Anti-inflammatory effects of YGQ leaves extracts on TNF-α and IL-6 in LPS-stimulated macrophages

**Fig. 1:**
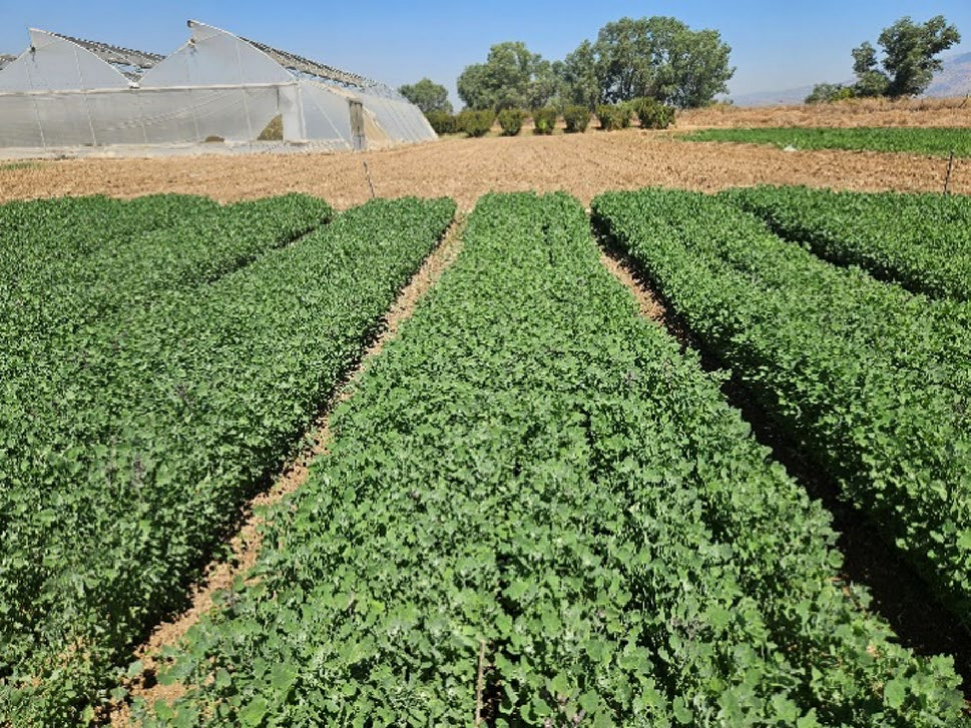
Young green quinoa. A representative photograph of young green quinoa grown at the research farm before harvest.

**Fig. 2:**
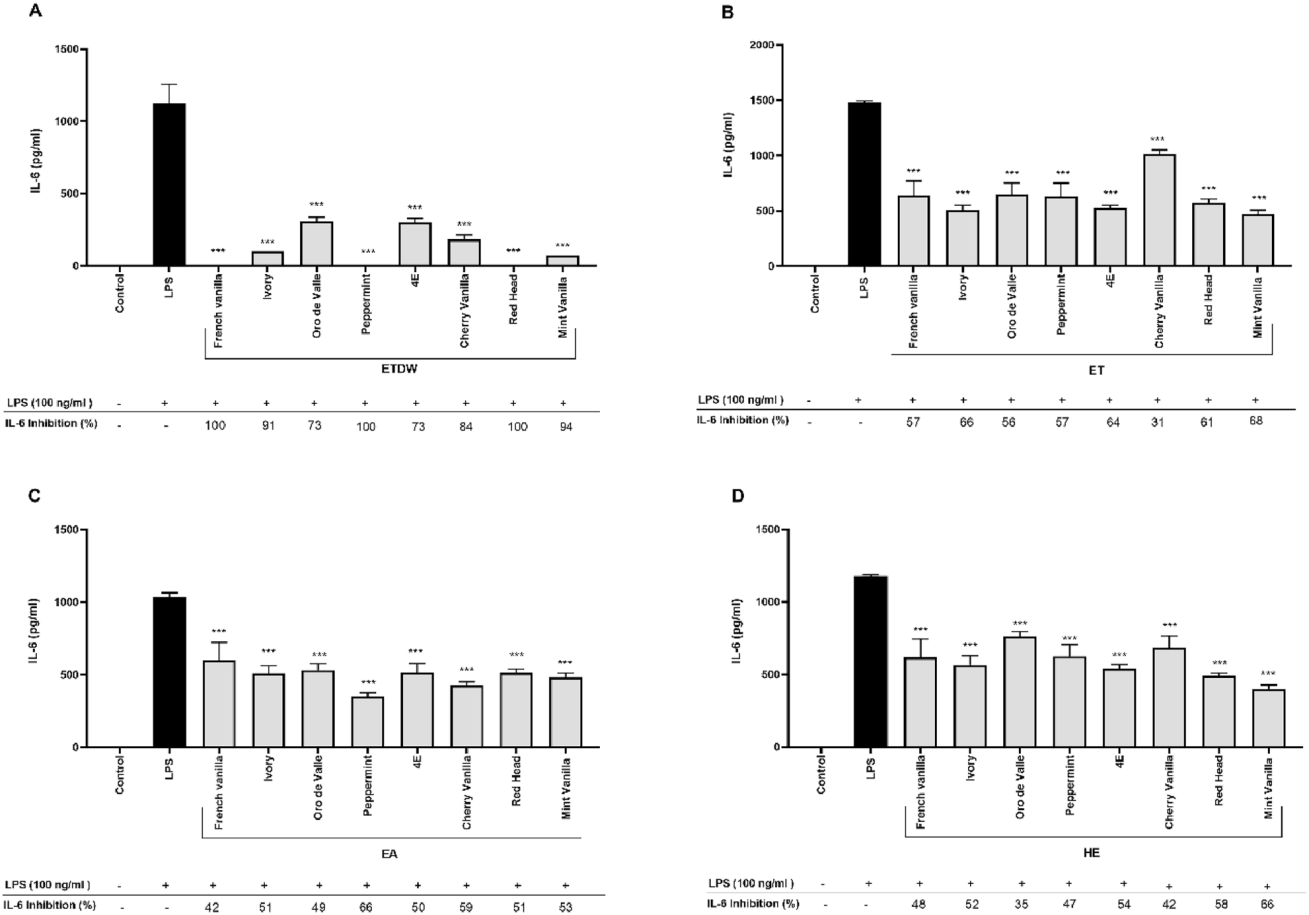
The effect of eight quinoa accession leaves extracts on LPS-induced IL-6 secretion in RAW264.7 cells. The macrophage cell line RAW264.7 was incubated with eight quinoa extracts for 30 min, followed by stimulation with 100 ng/ml LPS for 4 h. A control group was treated with DMSO only, while another control group was treated with LPS only. The figure is divided into four parts (A, B, C, and D), representing the effect of ETDW, ET, EA, and HE extracts, respectively, on LPS-induced IL-6 secretion. Data are presented as the mean ± SD of triplicate technical measurements from four independent experiments (n = 4). A one-way ANOVA was performed to assess statistical significance, assuming a normal distribution, with multiple comparisons corrected using Tuckey’s test. A significant reduction in IL-6 levels was observed compared to the LPS-only control group (*p* < 0.001).

Several YGQ-leaves extracts of different quinoa accessions, that were grown in the Gadash farm (Fig. 1) under the same conditions, were produced and optimized to find the optimal concentration that can inhibit the secretion of pro-inflammatory cytokines. It was found that the highest activity in inhibiting cytokine secretion was obtained at a concentration of 50 μg/ml. Thus, this concentration was selected as the optimum for determining the inhibitory activity of the various quinoa extracts on TNF-α and IL-6 production in LPS-activated RAW264.7 macrophage cells. The presence of each quinoa extract inhibited IL-6 production. However, to varying extents and with different effects, ETDW extracts showed the best inhibitory activity on IL-6 secretion (Fig. 2A and Table 1). The ETDW extracts from eight accessions tested had inhibitory percentages ranging from 73 to 100%, with French Vanilla, Peppermint, and Red Head, showing the highest inhibition (100%). The ET extracts (Fig. 2B and Table 1) also had inhibitory percentages ranging from 31 to 68%, with Mint Vanilla showing the highest inhibition (68%). The EA extracts (Fig. 2C and Table 1) had inhibitory percentages ranging from 42 to 66%, with Peppermint showing the highest inhibition (66%). The HE extracts (Fig. 2D and Table 1) also had inhibitory percentages ranging from 35 to 66%, with Mint Vanilla showing the highest inhibition (66%).

**Table 1:** Inhibition of IL-6 and TNF- α production in LPS-activated macrophage cells by eight YGQ accession of four extractants (50 μg/ml).

Extract	Quinoa Accession	Extract yield (%)	IL-6 inhibition (%)	TNF-a inhibition (%)	Cytotoxicity test
ETDW	French Vanilla	7	100	8	–
	Ivory	9	91	–	–
	Oro de Valle	9	73	–	–
	Peppermint	8	100	–	–
	4E	9	73	–	–
	Cherry Vanilla	10	84	–	–
	Red Head	11	100	–	–
	Mint Vanilla	9	94	6	–
ET	French Vanilla	5	57	7	–
	Ivory	5	66	–	–
	Oro de Valle	5	56	9	–
	Peppermint	6	57	–	–
	4E	7	64	–	–
	Cherry Vanilla	5	31	7	–
	Red Head	5	61	15	–
	Mint Vanilla	6	68	–	–
EA	French Vanilla	3	42	3	–
	Ivory	3	51	–	–
	Oro de Valle	3	49	–	–
	Peppermint	3	66	12	–
	4E	4	50	–	–
	Cherry Vanilla	3	59	6	–
	Red Head	3	51	9	–
	Mint Vanilla	4	53	4	–
HE	French Vanilla	2	48	22	–
	Ivory	3	52	20	–
	Oro de Valle	2	35	16	–
	Peppermint	3	47	4	–
	4E	3	54	7	–
	Cherry Vanilla	4	42	–	–
	Red Head	4	58	20	–
	Mint Vanilla	3	66	14	–

Analysis of statistical significance using one-way ANOVA revealed that the crude extracts had a significant *p* value of *p* < 0.001. This indicates a highly significant inhibition of IL-6 secretion by quinoa leaves extracts, suggesting a strong and reproducible biological effect. IL-6 plays a leading role in the regulation of inflammatory responses and is implicated in the pathogenesis of chronic inflammatory diseases, including rheumatoid arthritis, inflammatory bowel disease, and cardiovascular disorders. Elevated IL-6 levels contribute to prolonged immune activation and tissue damage, making it a key therapeutic target in inflammation-related conditions^26^. The ability of quinoa leaves-based extracts to significantly suppress IL-6 secretion in LPS-activated macrophages highlights their potential as a natural anti-inflammatory agent.

**Fig. 3:**
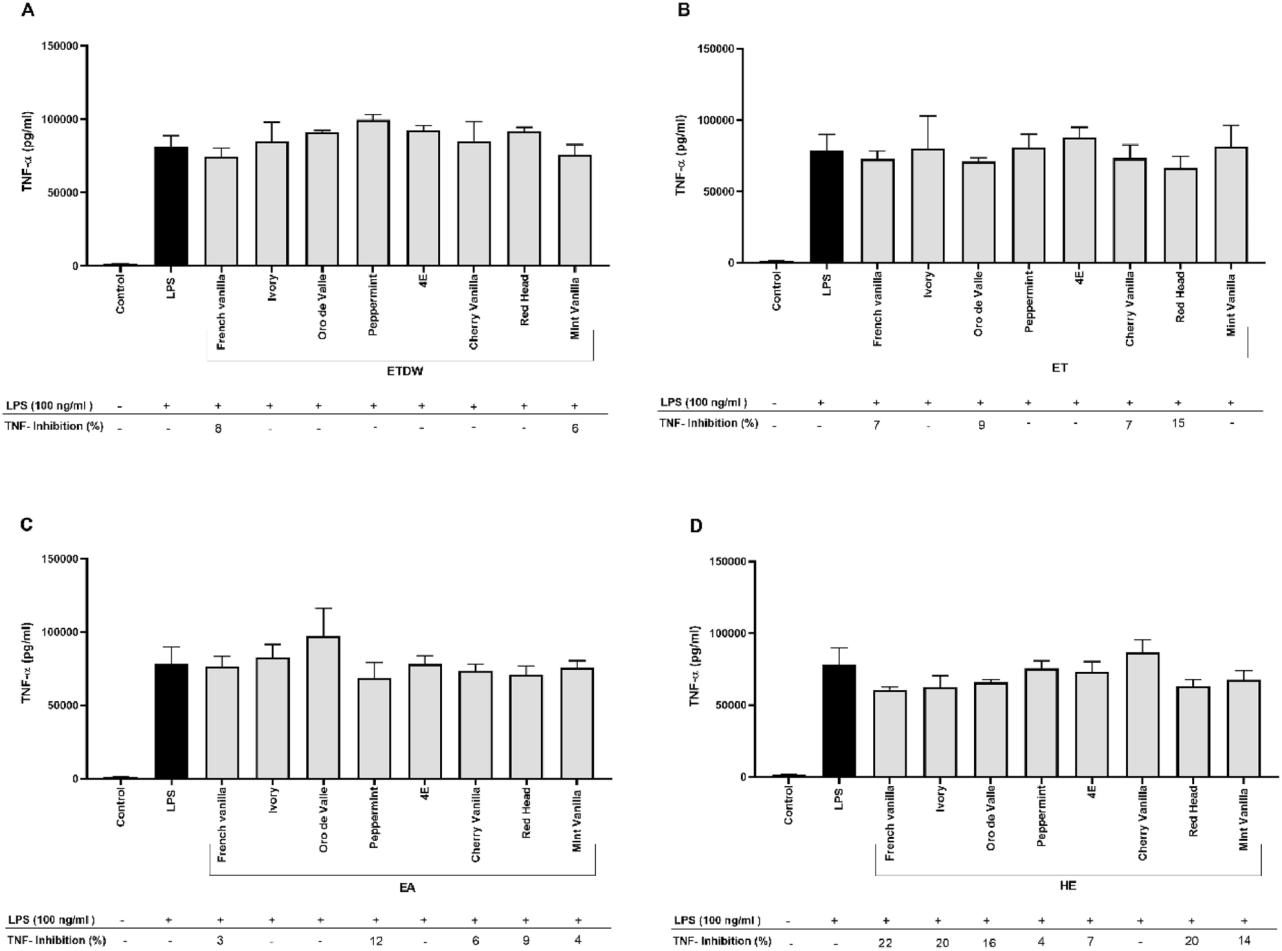
The effect of eight quinoa accession leaves extracts on LPS-induced TNF-α secretion in RAW 264.7 cells. The macrophage cell line RAW 264.7 was incubated with eight quinoa accession-based extracts for 30 min, followed by stimulation with 100 ng/ml LPS for 4 h. A control group was treated with DMSO only, while another control group was treated with LPS only. The figure is divided into four parts (A, B, C, and D), representing the effect of ETDW, ET, EA, and HE extracts, respectively, on LPS-induced TNF-α secretion. Data are presented as the mean ± SD of triplicate technical measurements from four independent experiments (n = 4). A one-way ANOVA was performed to assess statistical significance, assuming a normal distribution, with multiple comparisons corrected using Tuckey’s test. The results are presented as a percentage of inhibition relative to the LPS control group.

**Fig. 4:**
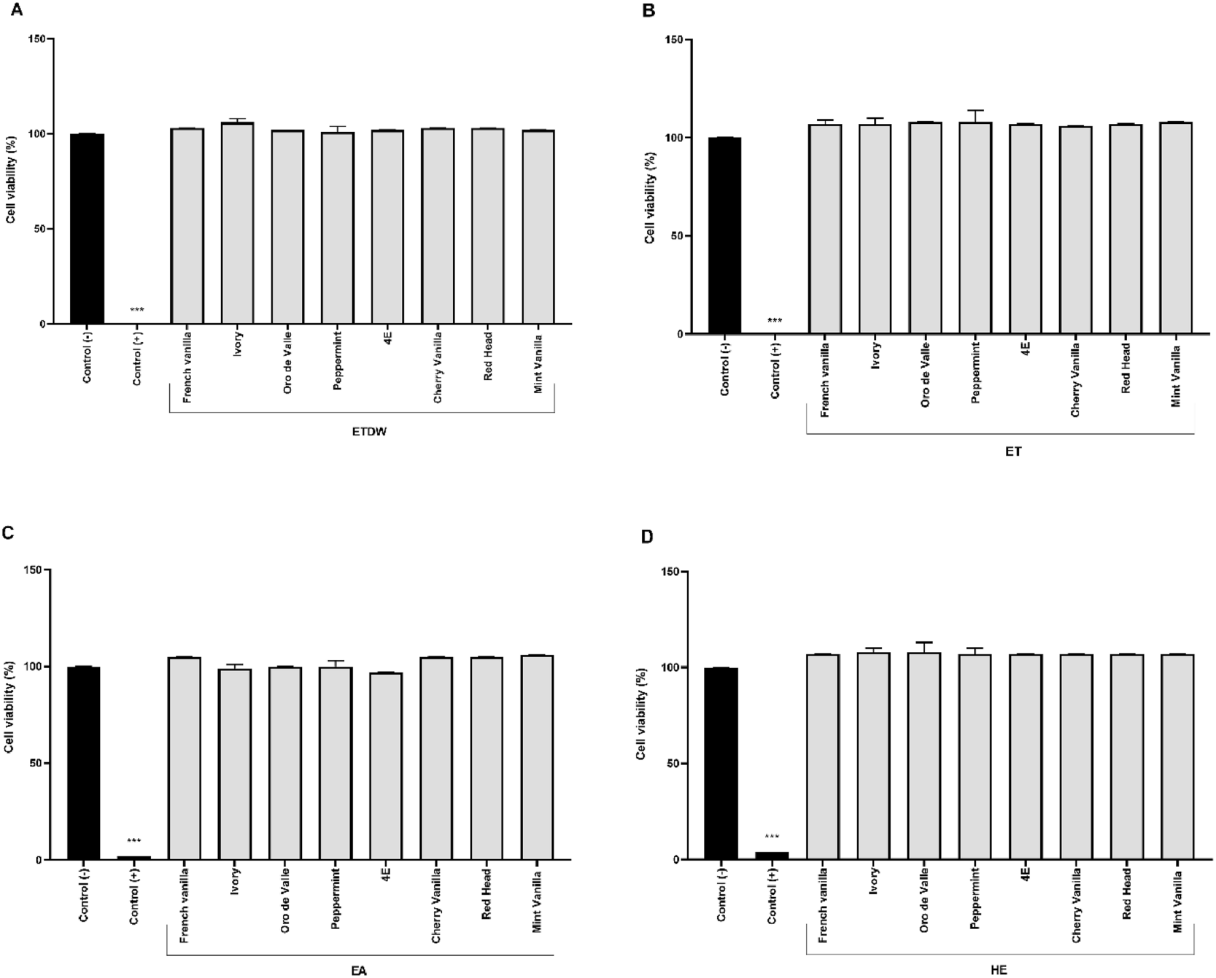
Cytotoxicity test on mouse macrophage RAW 264.7 cells using quinoa leaves extracts. The macrophages were treated with quinoa extracts for 4h, and the toxicity was determined using a WST-8 assay. The figure is divided into four parts, labeled A, B, C, and D, which show the results of ETDW, ET, EA, and HE extracts, respectively. The test was conducted with an untreated cell group as a negative control (control (−)), and non-stimulated cells were used as a positive control. Cell death control was also performed using cells treated with 10 μL of 1% Triton-X (control (+)).

The statistical analysis using one-way ANOVA revealed a highly significant effect of the crude extracts on IL-6 inhibition (*p* < 0.001), indicating that the observed reduction in IL-6 levels is unlikely to be due to random variation. Interestingly, the results of TNF-α cytokine inhibition (Fig. 3, Table 1) showed that none of the extracts inhibited the secretion of TNF-α. To rule out non-specific or pro-inflammatory activity, macrophages were also incubated with the crude quinoa extracts without LPS. The results showed that the extracts did not affect the secretion of either IL-6 or TNF-α (Figures S1 and S2). The effect of YGQ leaves-based extracts on the viability of RAW264.7 macrophages was also evaluated using WST-8 assays. The assays were performed on cells incubated with quinoa extracts for 4h. The results showed that all extracts did not affect cell viability at a concentration of 50 μg/mL (Fig. 4 and Table 1).

### Comparative LC–MS/MS metabolic profile of eight quinoa accession leaves-based extracts

**Fig. 5:**
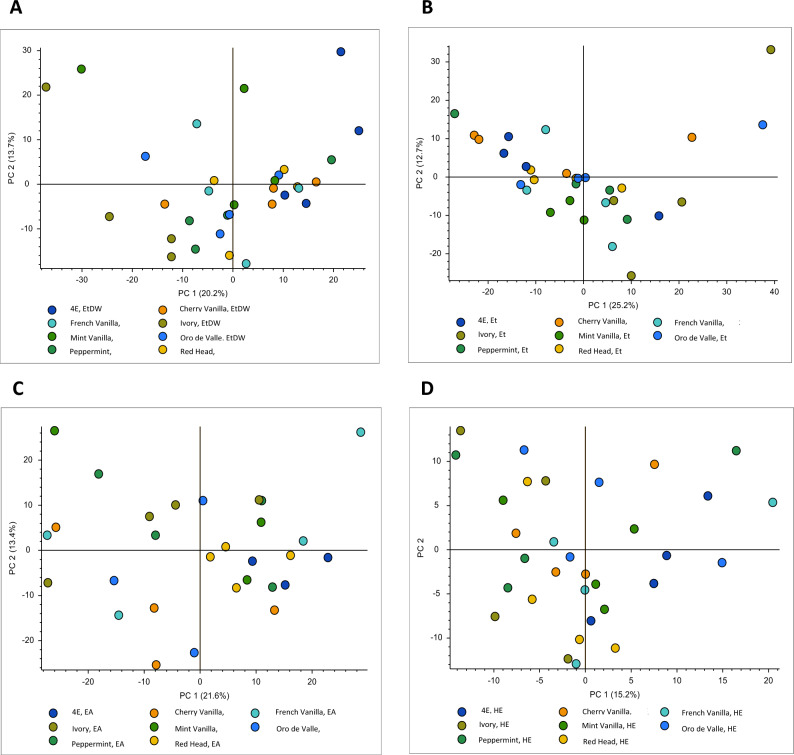
PCA analysis represents the overall variance between the metabolites identified in the extracts of leaves from eight quinoa accessions. PCA is a technique used to reduce the dimensionality of data while preserving as much information as possible. The figure is divided into four parts labeled A, B, C, and D, representing the overall variability of metabolites in ETDW, ET, EA, and HE extracts, respectively. The first and second principal components, PC1 and PC2, are plotted on the graph. PC1 represents the direction of the most significant variation in the data, while PC2 represents the second most significant direction, orthogonal to PC1.

The PCA analysis on the metabolic profile of eight quinoa accessions, which included 1300 metabolites, was performed using LC–MS/MS. The results indicate that the extracts from the four different solvents (ETDW, ET, EA, and HE) exhibited similar variability between the metabolites (Fig. 5A–D). The two main components in the ETDW extract explained 33.9% of the total variance, with 20.2% attributed to PC1 and 13.7% to PC2. Similarly, the two main components in the ET extract explained 37.9% of the total variance, the EA extract 35%, and the HE extracts 27.1%. The PCA results (Fig. 5, Table S1) suggest that the metabolic profiles of different quinoa accessions exhibit substantial overlap, indicating a high degree of similarity in metabolite composition across extracts suggesting that the quinoa accession type did not influence the metabolite profiles in the extracts. This result could indicate that certain metabolites are commonly present, reflecting conserved metabolic pathways shared across all accessions, or it may suggest a low genetic diversity in the accessions’ metabolic composition used in this study. These findings have important biological implications, as they imply that quinoa accessions exhibit similar metabolic characteristics, which could impact agricultural practices, breeding strategies, and the optimization of quinoa-derived extracts for specific bioactive compounds.

**Fig. 6:**
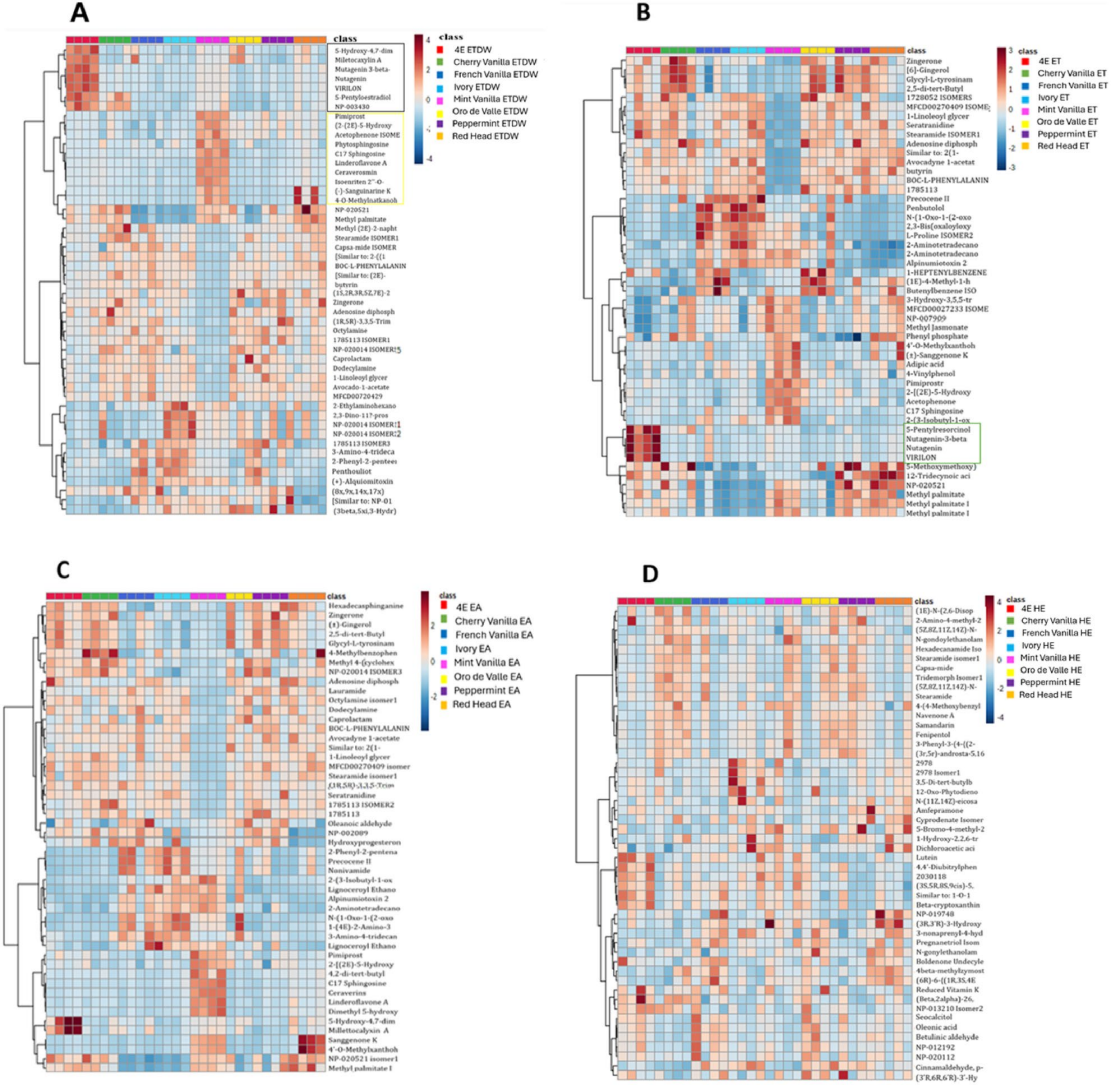
Heat map illustrating the distribution of metabolites among eight quinoa accessions, with different solvents used in the extraction process. The figure is divided into four parts, labeled A, B, C, and D, representing the metabolite profile of ETDW, ET, EA, and HE extracts, respectively. The heat maps were generated based on the concentrations of potential candidate metabolites obtained through univariate analysis. The color scale of the map represents an increase in the relative concentration of each metabolite as red and a decrease in concentration as blue. The names of the metabolites are listed on the right side of each row, and the eight quinoa accessions are shown in the legend.

A heat map was used to illustrate the metabolite distribution among the eight quinoa accessions extracted using different solvents. By analyzing the heat map, we can determine how each metabolite differs among the different accessions and extracts. When extracted using ETDW, the two varieties, 4E and Mint Vanilla, differed from the other accessions in metabolite content. Specifically, 4E had a group of metabolites marked with a black square, while Mint Vanilla had a group marked with a yellow square (Fig. 6A). In contrast, the metabolic distribution was similar among the other accessions. In the ET extract (Fig. 6B) and the EA extract (Fig. 6C), there was almost no discernable difference between the accessions regarding metabolite content, except for the Mint Vanilla ET, EA, and 4E ET extracts. The 4E ET extract contained a group of metabolites not found in the other accessions, marked with a green square (Fig. 6B). No scientific studies have been found documenting the biological activity of Nuatigenin or VIRILON. A review of the scientific literature did not reveal any experimental data or findings indicating the physiological or pharmacological effects of these compounds. Interestingly, in the Mint Vanilla ET and EA extracts (Fig. 6A,C), we observed a compound profile lacking metabolites that exist in the other accessions, such as zingerone which is known for its anti-inflammatory properties^27^, though it did not affect the biological activities that showed the same inhibitory effect as other accessions (Table 1), suggesting that these metabolites are most probably not responsible for its anti-inflammatory activity. On the other hand, the Mint Vanilla ET and EA metabolite profile include methyl palmitate which is known as an anti-inflammatory agent^28^. No detectable metabolite difference was observed when comparing the eight accessions within the HE extracts (Fig. 6D).

**Fig. 7:**
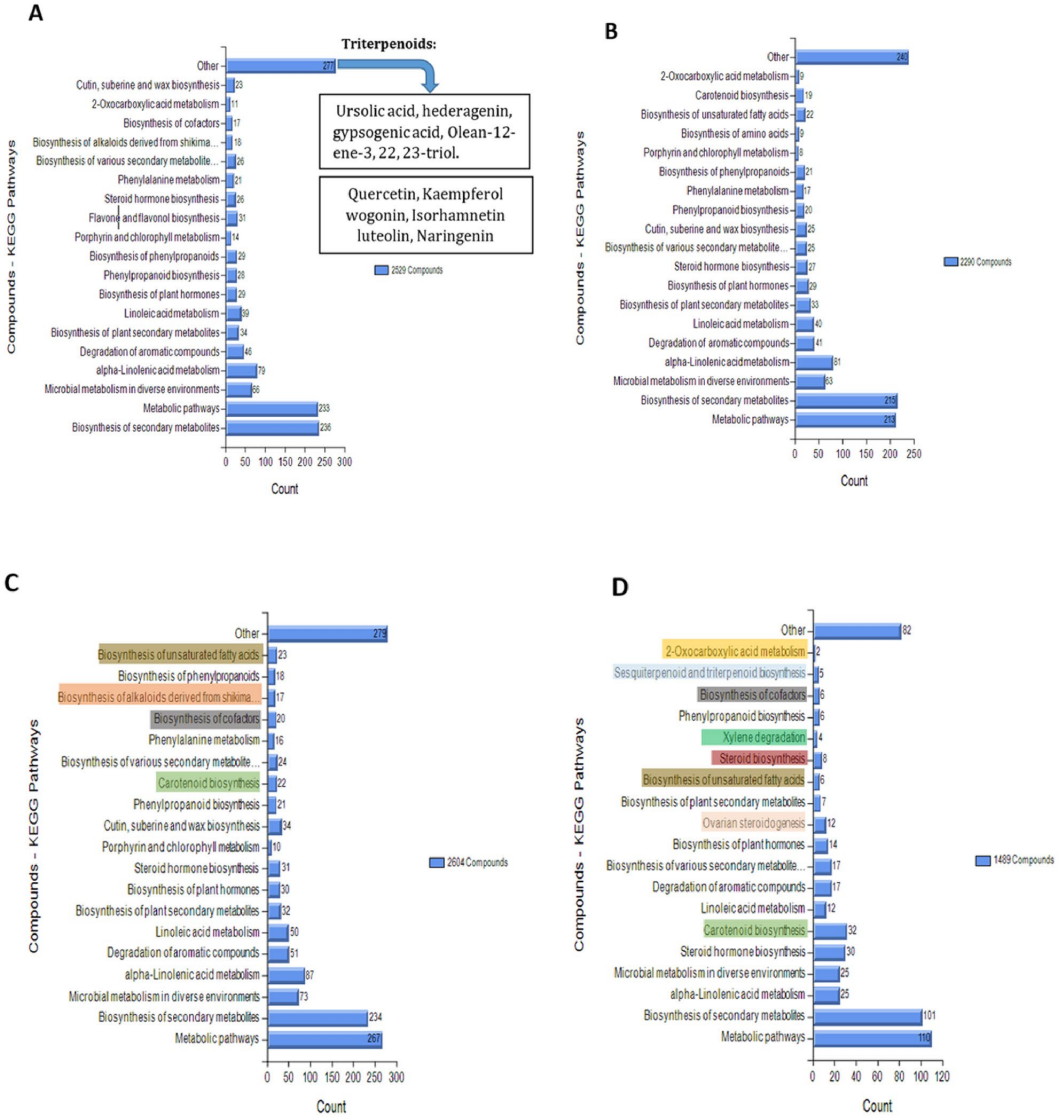
KEGG pathway classification statistics of the metabolites identified in the extracts of eight quinoa accessions. The histogram is divided into four parts, labeled A, B, C, and D, representing the KEGG metabolic pathways of ETDW, ET, EA, and HE extracts, respectively. The ordinate represents the names of KEGG metabolic pathways, while the abscissa shows the number of materials annotated in each pathway. The histogram uses color to indicate the unique metabolic pathways of a particular solvent. Source: KEGG Pathway Database^29–31^.

A KEGG pathway graph adapted from the KEGG Pathway Database^29–31^ was used to represent the different metabolic pathways to which the metabolites of the eight quinoa accessions belong when extracted using different solvents. Figure 7 illustrates these pathways for ETDW, ET, EA, and HE extracts. The KEGG graph showed that only in the ETDW extract (Fig. 7A) was a metabolic pathway called "Flavone and flavanol biosynthesis" identified, which does not appear in the other extracts. Flavonoids, such as Quercetin, Kaempferol, Wogonin, Isorhamnetin, Luteolin, and Naringenin, belong to this metabolic pathway and have been known to have anti-inflammatory effects. This extract discovered a family of substances that can be linked to the metabolic pathway “other”. This family includes triterpenoid compounds, such as saponins, like Ursolic acid, Hederagenin, and Gypsogenic acid, which are widely documented in quinoa and are known to have anti-inflammatory effects.

In contrast, Fig. 7B–D (ET, EA, and HE extracts) show a metabolic pathway called “Carotenoid biosynthesis” that does not exist in the ETDW extract. This pathway is related to the production of carotenoids, a group of pigments with health benefits found in plants. Overall, the KEGG pathway graph represents the different metabolic pathways among eight quinoa accessions when extracted using different solvents and highlights the presence of anti-inflammatory compounds in the ETDW extract.

The findings from PCA, heatmap, and KEGG pathway analyses provide crucial insights into the metabolite composition of quinoa accessions and support the hypothesis that YGQ leaves are a rich source of bioactive compounds, particularly those with potent anti-inflammatory properties. PCA analysis revealed that the overall metabolite profiles across the eight quinoa accessions exhibited a high degree of similarity, suggesting that the bioactive compounds present in these leaves are relatively consistent. However, heatmap analysis highlighted distinct differences in metabolite distribution within the extracts, particularly in the ETDW extract, where accessions 4E and Mint Vanilla displayed unique metabolite profiles. Notably, specific metabolites such as phytosphingosine and certain saponins were differentially present in particular accessions. KEGG pathway analysis further reinforced these findings by identifying key biological pathways influenced by these metabolites. For example, two metabolite families, flavonoids and triterpenoids, were only present in the ETDW extract. Moreover, the study results demonstrated that the most significant inhibition of IL-6 secretion was observed in the ETDW extract, suggesting that these bioactive compounds are likely responsible for the higher observed anti-inflammatory effects. Despite some differences in metabolite content between accessions, the overall similarity in metabolite profiles suggests that quinoa leaves, regardless of accession, maintain a stable composition of bioactive compounds capable of exerting anti-inflammatory effects.

## Discussion

Given the rising prevalence of NCDs associated with chronic inflammation, integrating young green quinoa (YGQ) extracts into dietary products could contribute to mitigating inflammation-related health conditions, such as cardiovascular disease, metabolic disorders, and autoimmune diseases. Functional foods incorporating YGQ extracts could be formulated in various ways, including fortified beverages, nutritional supplements, health bars, or meal replacements aimed at promoting anti-inflammatory diets. The high nutritional content of YGQ leaves, including proteins, fiber, vitamins, and bioactive compounds, further enhances their potential as a plant-based functional food supporting immune function and overall well-being.

**Fig. 8:**
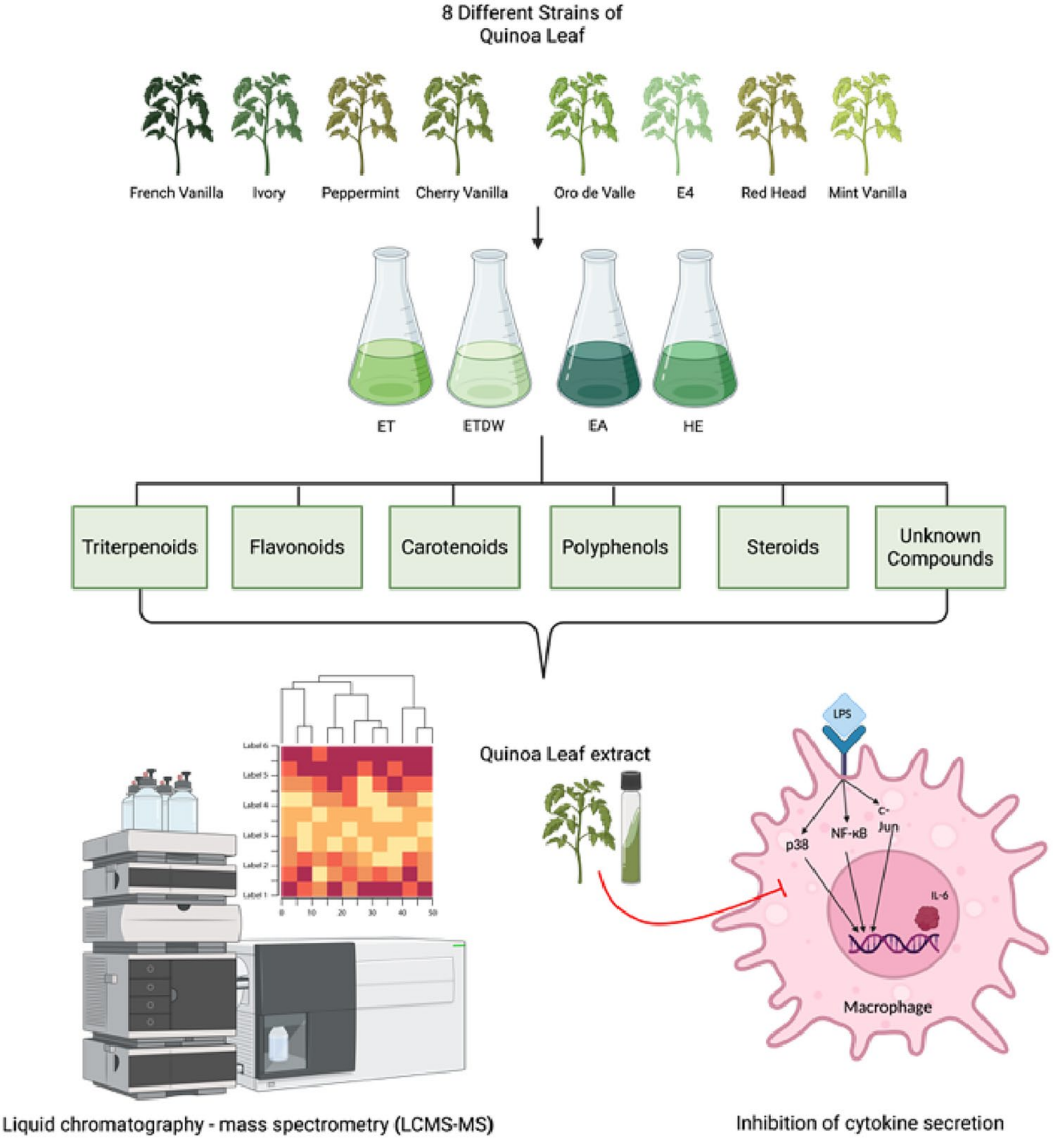
An overview of the research pipeline applied in this study.

This study examined the impact of extracts from eight YGQ accessions on the secretion of cytokines integrating field study, metabolomics, and immune modulation experiments (Fig. 8). The results of this study show that YGQ leaves-based extracts at a dose of 50 μg/ml can specifically reduce the excessive release of IL-6 from LPS-activated mouse macrophages without toxic effect (Figs. 2, 4, and Table 1) and up to 100% inhibitory effect. Interestingly, there was no inhibition in the secretion of the cytokine TNF-α (Fig. 3 and Table 1). Additionally, when the extracts were tested on cells without LPS, no secretion of either cytokine was found, indicating no nonspecific or pro-inflammatory activity (S1, S2). The significant inhibitory effect of the extracts of the eight quinoa accessions on the secretion of the cytokine IL-6, as opposed to the insignificant effect on the release of the cytokine TNF-α, leads us to assume that YGQ leaves extracts can serve as specific IL-6 suppressors and that the delay percentages obtained are not the result of cell death. Despite previous studies on quinoa grains showing similar anti-inflammatory effects, specifically a decrease in the secretion of pro-inflammatory cytokines TNF-α and IL-6^32^, no previous studies have been found on the effects of quinoa leaves. These profound and significant results could be valuable for conditions characterized by excessive IL-6 production, such as rheumatoid arthritis and other chronic inflammatory diseases^33^. However, further studies are necessary to determine whether the extracts exhibit comparable efficacy to existing IL-6 inhibitors and to elucidate their mode of action. Additionally, preclinical validation in in vivo models is essential to assess bioavailability, efficacy, and potential toxicity before considering its functionality in NCDs and their potential applications in functional food development or adjunctive therapies for inflammatory diseases.

In addition, PCA analysis was employed as a valuable tool to identify differences between the eight accessions of YGQ- leaves’ metabolites. The results of the PCA (Fig. 5) showed a similarity between the accessions’ content of metabolites present in each solvent. These results are consistent with the inhibition percentages obtained for the secretion of IL-6, as all eight accessions showed statistical significance for their inhibitory effect, and the inhibition percentages were similar across accessions.

This study also evaluated the variations of each metabolite in the different YGQ-based extracts using a heat map graph, which provides additional information on the differences between the eight quinoa accessions and complements the overall picture provided by the PCA graph. The heat map showed that metabolite distribution was generally similar across the different accessions for each solvent. However, some variations were observed, such as in the ETDW extract (Fig. 6A) where two accessions (4E and Mint Vanilla) had different metabolite content compared to the others, and in the ET extract (Fig. 6B) where accession 4E had a group of metabolites not found in other accessions. As all accessions were grown under the same conditions and harvested at the same time point, the results suggest that environmental factors had no influence. The metabolite profiles and bioactivity data indicate that the extraction solvent, rather than genetic variation among accessions, was the primary determinant of biological activity.

Furthermore, phytosphingosine was detected in Mint Vanilla (ETDW extract), which has the potential to affect the NF-κB or the JAK/STAT^34^. This substance may influence the inhibition of IL-6. Moreover, in other extracts, (ET, EA) (Fig. 5B–D, respectively), the distribution of the metabolites of Mint Vanilla was slightly different from the others, but the inhibitory percentages of this variety were like those of the other accessions. Although the accessions have different metabolites, they have similar activity to inhibit the secretion of IL-6, suggesting that a different composition of substances can have similar effects.

Different extractants significantly influence the metabolite profile due to their varying solubility properties, polarity, and affinity for specific compounds^35^. Polar solvents such as methanol and ethanol typically extract hydrophilic metabolites, including polyphenols and flavonoids, which are known for their anti-inflammatory properties^36^. In contrast, non-polar solvents like chloroform and hexane preferentially extract lipophilic metabolites such as terpenoids and certain alkaloids^37^. For example, methanol has been shown to efficiently extract compounds like quercetin and kaempferol, both of which are recognized for their ability to modulate pro-inflammatory cytokine production, including IL-6. In this study, comparative LC-MS/MS analysis demonstrated that each extractant resulted in a distinct metabolite profile. Ethanol: water (ETDW) extracts were enriched with flavonoids, such as quercetin and kaempferol, which are well-documented for their anti-inflammatory effects and were associated with the highest IL-6 inhibition observed. Conversely, hexane extracts primarily contain lipophilic compounds such as carotenoids, which did not exhibit significant IL-6 inhibition. These findings highlight a clear relationship between extractant polarity and metabolite composition, directly influencing the extracts’ biological activity.

Moreover, this study analyzed the metabolites of quinoa leaves extracts using a Pathway KEGG graph, which summarizes the metabolites and associates them with different metabolic pathways. The KEGG graph’s results showed that two families of metabolites appear exclusively in the ETDW extract (Fig. 7A). The first family of metabolites is called flavonoids, a large group of components called polyphenols. Flavonoids have anti-inflammatory activity mediated through various mechanisms, such as the release inhibition of several pro-inflammatory cytokines and antioxidants^25^. A study on quinoa leaves published by Gawlik-Dziki et al.^9^ discovered four flavonoids (Quercetin, Kaempferol, Isorhamnetin, and Routine) known to have anti-inflammatory abilities. These flavonoids inhibit the activation of transcription factors, such as STAT-1 and NF-κB, which release pro-inflammatory cytokines such as IL-6^25^. Flavonoids have been shown to inhibit pro-inflammatory cytokines such as IL-6^38^, thus flavonoids identified in the present study, may be significant contributors to the observed IL-6 inhibition. The presence of these flavonoids in YGQ leaves further highlights the potential of YGQ as a source of natural anti-inflammatory agents. Since quercetin and kaempferol were found only in the ETDW extract, we hypothesize that one of these compounds is responsible for the higher inhibition observed in the ETDW extract compared to the other extracts. In addition, Gawlik-Dziki et al., discovered several other flavonoids are present in quinoa leaves that were not previously known in the literature. One of these substances is wogonin, which inhibits the activation of the STAT-1 transcription factor responsible for the unique release of IL-6^39^. Another discovery is the triterpenoid family (usually saponins). Saponins give leaves a bitter taste and are known as "anti-nutritional" substances as they bind to various minerals and inhibit their absorption. However, there is growing evidence that saponins can have beneficial health effects, including anti-cancer, anti-inflammatory, and anti-hypocholesterolemia effects. An example of the saponins that were found in our study is hederagenin, which is involved in various anti-inflammatory effects^22^.

In previous studies focusing on quinoa grains, researchers investigated the biological activity of hederagenin on mouse macrophage cells. Their findings revealed that in the presence of fractions containing hederagenin, there was a notable decrease in the secretion of NO and various pro-inflammatory cytokines, such as TNF-α and IL-6^40^. Similarly, our research identified the presence of hederagenin in quinoa leaves, suggesting a potential similar effect to that observed in the grains. Another example of triterpenoids found in ETDW extract is uric acid, which is mediated by repression of the transcription factor NF-κB^41^. As mentioned, these families (flavonoids, triterpenoids) are only found in the ETDW extract, which leads us to hypothesize that one of the substances belonging to these families is responsible for the highest inhibition of IL-6 secretion compared to the other extracts. Furthermore, Carotenoids are present in the ET, EA, and HE extracts but are absent in the ETDW extracts (Fig. 6B–D). Carotenoids, well-known natural pigments, possess antioxidant, anti-apoptotic, and anti-inflammatory properties, as demonstrated in vivo and in vitro studies^42^. For instance, lutein, a Carotenoid found in these extracts, exhibits anti-inflammatory properties by inhibiting the NF-κB transcription factor^43^.

This study highlights YGQ leaves as a promising source of anti-inflammatory compounds, particularly IL-6 inhibitors, with potential applications in functional foods and sustainable agriculture. The presence of flavonoids, carotenoids, and triterpenoids in YGQ extracts likely contributes to its observed bioactivity. Comparative analyses suggest, for example, that quinoa leaves contain flavonoid levels similar to those found in spinach (Spinacia oleracea) and kale (Brassica oleracea var. sabellica), both known for their strong anti-inflammatory properties^44,45^. Quinoa leaves have been reported to contain significant amounts of flavonoids, with rutin concentrations reaching up to 914 mg/100 g^46^. This is similar to the high amounts found in other leavesy greens. For instance, kale contains between 661 and 892 mg/100 g of total flavonoids, including quercetin and kaempferol^47^. Spinach contains approximately 10 mg/g quercetin equivalents (equivalent to 1000 mg/100 g)^48^. These comparisons suggest that quinoa leaves are a potent source of flavonoids, which may contribute to their anti-inflammatory properties. Integrating our findings from PCA, heatmap, and KEGG pathway analyses emphasizes that quinoa leaves can serve as a reliable source of bioactive compounds with anti-inflammatory properties. This comprehensive approach not only contextualizes the observed IL-6 inhibition but also confirms the potential of quinoa leaves as functional ingredients for the development of foods targeting chronic inflammatory diseases. Additionally, these results highlight quinoa leaves as promising candidates for sustainable food innovations that may contribute to improved human health and food security.

To better understand the significance of YGQ leaves as a functional ingredient that could be incorporated into commercially viable products, future research should include for example in vivo studies for bioavailability and absorption. Maintaining the bioactive properties of YGQ extracts during food processing, storage, and formulation is crucial as heat, pH, and other food-processing factors could degrade key compounds, potentially reducing their efficacy. Encapsulation technologies or novel food preservation methods may be required to maintain their functional benefits. While quinoa grains are widely recognized as a healthy food, YGQ leaves are relatively unknown to consumers. Effective consumer education and marketing strategies will be necessary to build awareness of their health benefits and create demand. Functional foods with health claims must comply with regulatory requirements set by authorities such as the FDA (U.S.), EFSA (Europe), and other international agencies. Establishing safety, efficacy, and standardized dosages for YGQ extracts will be essential for market approval.

In addition, environmental factors such as soil type, climatic conditions, and the growth stage of quinoa plants can significantly affect the concentration and composition of bioactive compounds. Large-scale cultivation and sustainable harvesting of YGQ leaves must be optimized to ensure consistent supply. Despite their potential, YGQ leaves have received limited attention to support sustainable food chain. From an economic perspective, quinoa leaves have the potential to be developed into value-added products for the food and pharmaceutical industries. Their rich nutritional and bioactive compound profile makes them a promising resource for functional food development. However, their commercial viability depends on factors such as consumer demand, processing costs, and market accessibility. Further research is needed to assess whether integrating quinoa leaves into existing value chains can provide financial benefits for farmers while reducing agricultural waste.

In terms of agronomic feasibility, YGQ has a short cultivation and harvesting period with multiple cycles with the ability to grow in harsh conditions (high salinity, low water). However, optimizing harvesting techniques to ensure efficient leaves collection without compromising grain yield would be essential for sustainable implementation.

By addressing these considerations, quinoa leaves could transition from a discarded byproduct to a sustainable and economically viable resource, contributing to circular agriculture and food security. Developing cost-effective farming and processing methods will be critical for commercial feasibility.

To overcome these challenges, future research should focus on human trials, exploring optimal delivery methods for bioactive compounds, and developing scalable, sustainable production processes. Collaborations between scientists, food technologists, and industry stakeholders will be essential to bringing YGQ-based functional foods to the market.

This research provides a promising preliminary basis for prioritizing YGQ leaves as a sustainable candidate for further in-depth phytochemical, and anti-inflammatory research, NCDs, and crop research. Our findings highlight the phytochemical potential of YGQ leaves and their relevance in anti-inflammatory research. Leveraging the metabolomic data obtained, future studies should focus on establishing direct cause-effect relationships between YGQ bioactive compounds and their therapeutic effects, particularly in the context of NCDs.

## Materials and methods

### Reagents

RAW 264.7 cells, murine macrophage cell line, were purchased from the American Type Culture Collection (ATCC, USA). Additionally, Dulbecco’s Modified Eagle Medium (DMEM) was purchased from ATCC. Fetal bovine serum (FBS), penicillin–streptomycin, and glutamine were purchased from Biological Industries (Beit HaEmek, Israel). ELISA kits were purchased from PeproTech. LPS was purchased from Sigma-Aldrich (Sigma-Aldrich, Israel). WST-8 assay (Cell Counting Kit-F, Enzo Life Sciences Inc).

### Plant materials and agrotechnical management

Quinoa cultivation was conducted from mid-June to July 2020 in northern Israel at the `Gadash` research farm (altitude 71 m a.s.l., 33° 17′ 95″ N 35° 58′ 32″ E). Eight commercially available quinoa accessions were acquired from Wild Garden Seed (Oregon, US): French Vanilla, Ivory, Peppermint, Oro de Valle, Cherry Vanilla, Red Head, and Mint Vanilla. An additional accession, 4E, was acquired from Equinom Ltd. (Givat Brenner, Israel). In mid-June 2020, quinoa seeds of each accession were sown into 5 m^2^ plots with six rows (18 cm between rows) on each plot, at a planned density of 240 seeds m^-2^. Plots were randomized, with four plots for each quinoa accession. Seeds sprouted in plots, and plants were grown by irrigation using sprinklers at a rate of 1500 m^3^ ha^−1^. All plots received a basic fertilizer of 92 kg N ha^−1^ as urea (46% N). Plants were harvested 42 days after sowing when the dry matter percentage reached an average of 12%. Actual plant density at harvest stood at an average of ~ 150 plants m^−2^.

### Preparation of crude extracts

The leaves from each accession were dried and ground to a powder, and four different types of extracts were prepared: Ethanol: Water (ETDW) in a ratio of 70%:30% respectively, Ethanol (ET), Ethyl acetate (EA), and Hexane (HE) by stirring 5gr of leaves powder in 50ml solvent in 1:10 ratio for two hours at room temperature. Then, the liquid was filtered, the solvents were evaporated, and samples were dried using nitrogen. Each quinoa extract was dissolved in DMSO, to determine the inhibition of TNF-α and IL-6 production in LPS-activated RAW 264.7 macrophage cells by in vitro bioassay. The final concentration of DMSO in the culture medium was 0.1%, which is known to be non-toxic to the cells used in this study and does not interfere with cell viability, ensuring that the observed effects are attributable to the bioactive compounds rather than the solvent^49,50^.

### Measurement of the secretions of pro-inflammatory from RAW264.7 macrophages

RAW 264.7 murine macrophages were cultured in Dulbecco’s Modified Eagle Medium (DMEM) with high glucose, 10% fetal bovine serum (FBS), and 1% Penicillin streptomycin. Cells were seeded into a 75-cm^2^ flask at 37 °C in a humidified atmosphere with 5% CO2. Afterward, RAW 264.7 cells (1 × 10^5^ cells/well) were inoculated into a 96-well plate and cultured for 48h. During the experiment, macrophages were incubated with the quinoa extracts in the presence or absence of LPS. Various quinoa extracts were added 30 min before LPS (100 ng/mL) stimulation for an additional four hours of culturing. The amounts of TNF-α and IL-6 secreted from murine macrophage cells were measured with TNF-α and IL-6 ELISA kits (PepRotech) according to manufacturer instructions. The concentrations of the cytokines were measured using ELISA reader infinite M200 PRO (TECAN, Switzerland) at 450 nm with correction at 620 nm. In all experiments, the activity was compared to a standard steroidal anti-inflammatory treatment (dexamethasone), as a positive control of the cell response. The delay obtained was 40–60% in the various experiments, as is customary in the literature (data not shown)^51,52^.

### Cell viability (WST-8 assay)

The cytotoxicity of the macrophages treated with various quinoa extracts was determined using the WST-8 assay. Cells were grown in complete DMEM in 96-well plates (2 × 10^5^ cells/well) and incubated at 37 °C in an atmosphere of 5% CO2 for 48h. The assay was performed using samples and controls in triplicate. After 48h, we added 10 μl of quinoa extracts into the culture media in the plate and incubated for four hours. At the end of the four hours, 10 μL of WST-8 was added to each well and incubated for 1-4h. The untreated cells were assayed as a negative control. Non-stimulated cells were used as positive controls. Cell death control was performed using cells treated with 10 μL of 1% Triton-X. The absorbance of the samples was measured in a microplate reader spectrophotometer using 450 nm.

## Metabolomics profile of quinoa leaves extracts

The samples were analyzed by injecting 5 μL of the extracted solutions (at a concentration of 1000ppm) into a UHPLC connected to a photodiode array detector (Dionex Ultimate 3000), with a reverse-phase column (ZORBAX Eclipse plus C18, 3.0*100 mm, 1.8 μm). The mobile phase consisted of (A) DDW with 0.1% formic acid and (B) acetonitrile containing 0.1% formic acid. The gradient started with 2% B, then increased to 30% B in 4 min, then increased to 40% B in 1 min, and kept isocratic at 40% B for 3 min. Then increased to 98% B in 6 min and kept isocratic at 98% B for 9 min. Phase B returned to 5% in 3 min, and the column was allowed to equilibrate at 5% B for 5 min before the next injection. The flow rate was 0.4 mL/min. LC–MS/MS analysis was performed with a Heated Electrospray ionization (HESI-II) source connected to a Q Exactive™ Plus Hybrid Quadrupole-Orbitrap™ Mass Spectrometer Thermo Scientific™. ESI capillary voltage was set to 3500 V, capillary temperature to 300°C, gas temperature to 350 °C, and gas flow to 35 mL/min. The mass spectra (m/z 67-1000) were received in negative and positive-ion modes with high resolution (FWHM = 70,000). For MS2 analysis, collision energy was set to 15, 50, and 100 EVs. Peak determination and peak area integration were performed with Compound Discoverer 3.1 (Thermo Xcalibur, Version 3.1.0.305). Auto-integration was manually inspected and corrected if necessary. Compound identification relied on the McCloud database using MS2 data and the ChemSpider database using HRMS.

### Statistical analysis

The experimental data used in this study was collected from four field repeats and three separate experiments, which were conducted in triplicate. Statistical analyses were performed using GraphPad Prism. Data were analyzed using one-way ANOVA to compare differences among experimental groups. Post-hoc comparisons were conducted using Tukey’s multiple comparison test. Principal Component Analysis (PCA) was performed to assess the overall structure of the data, and heatmaps were generated to visualize the clustering of experimental groups based on their metabolic profiles. Results are presented as mean ± SEM, with *p* values < 0.05 considered.

## Conclusion

This study demonstrates, for the first time, that young green quinoa (YGQ) leaves-based extracts selectively inhibit IL-6 production in LPS-activated macrophages without affecting TNF-α, suggesting modulation of specific inflammatory pathways. Given the increasing concern over chronic inflammation and non-communicable diseases (NCDs), quinoa leaves emerge as a promising anti-inflammatory plant source with potential applications in functional foods. Unlike previous research focusing on quinoa grains, this study uniquely highlights the bioactive potential of YGQ in inflammation regulation.

The use of food-grade solvents ensures safety for human consumption, supporting the feasibility of incorporating quinoa leaves into nutraceuticals and health-promoting foods. This research also marks the first characterization of bioactive compounds in quinoa leaves extracts, paving the way for future studies on compound isolation and mechanistic insights. Moreover, as climate change and food security concerns rise, quinoa’s adaptability and nutritional value make it a compelling candidate for sustainable agriculture.

Ultimately, this study distinguishes quinoa leaves from quinoa grains, specifically identifying quinoa leaves as a potent source of IL-6 inhibition while quinoa grains show only moderate cytokine inhibitory effects^38^. The findings reinforce quinoa leaves as a potent and novel anti-inflammatory agent, warranting further exploration of their therapeutic and commercial potential.

## Supplementary Information

Supplementary Information.

## Data Availability

Data is provided within the manuscript or supplementary information files and will be open to the public upon acceptance.
