# Supplementary material for: Young green quinoa as a sustainable functional crop with anti-inflammatory effects in macrophage cells
Source: Sci Rep. 2025 Jun 26;15:20308. doi: 10.1038/s41598-025-03742-w (PMC12202808; doi:10.1038/s41598-025-03742-w)
Supplement: Supplementary file 1 — Supplementary Information. [file 41598_2025_3742_MOESM1_ESM.docx]

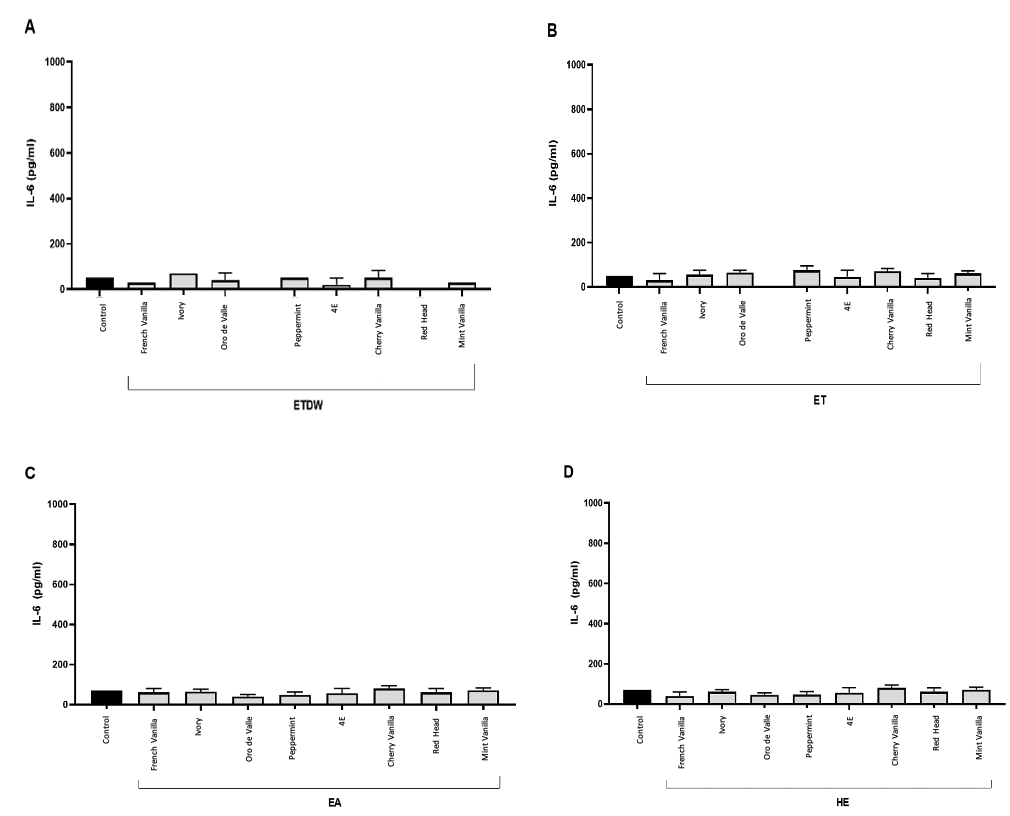


**Figure S1. The effect of eight quinoa varieties (grown under high-density conditions and harvested during the summer) extracts on induced Interleukin (IL-6) secretion in RAW 264.7 cells.**  The macrophage cell line RAW 264.7 was incubated with eight quinoa extracts (grown under high density and harvested in the summer) for 4h. A control group was treated with DMSO only. The figure is divided into four parts, labeled A, B, C, and D, which show the effect of ETDW, ET, EA, and HE extracts on inhibiting LPS-induced IL-6 secretion. The data is presented as the mean ± SD of triplicate measurements in four field repeats.


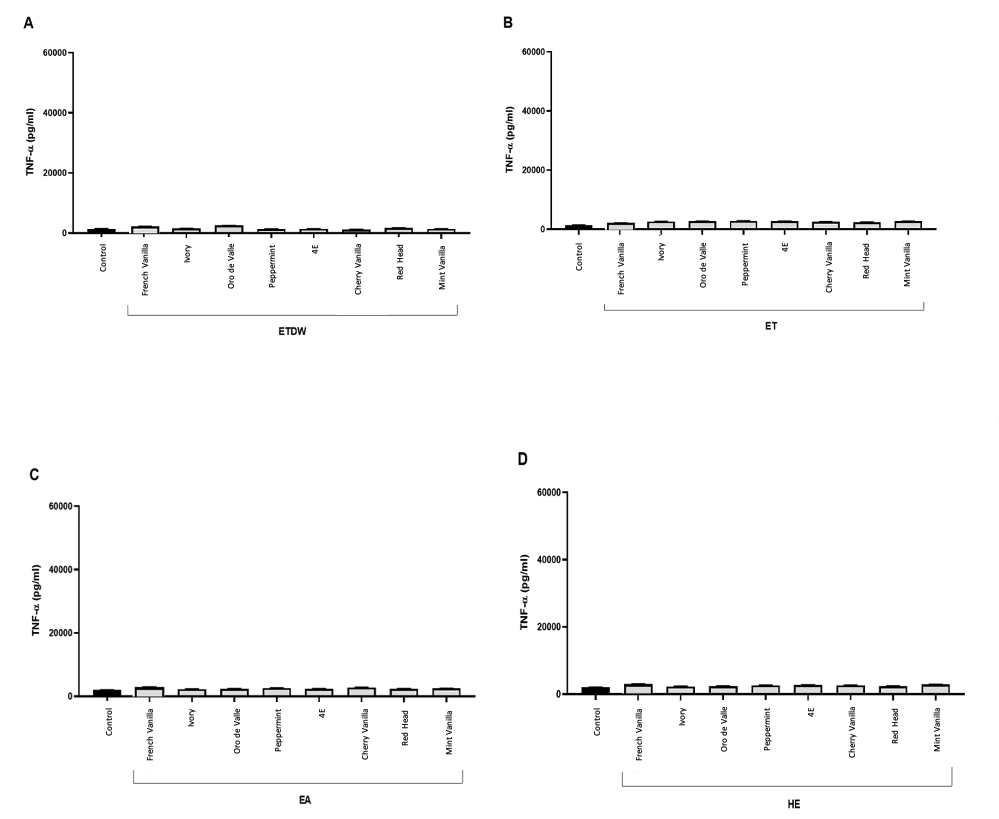


**Figure S2. The effect of eight quinoa varieties (grown under high-density conditions and harvested during the summer) extracts on induced Interleukin (TNF-α) secretion in RAW 264.7 cells.** The macrophage cell line RAW 264.7 was incubated with eight quinoa extracts (grown under high density and harvested in the summer) for 4h. A control group was treated with DMSO only. The figure is divided into four parts, labeled A, B, C, and D, which show the effect of ETDW, ET, EA, and HE extracts on inhibiting LPS-induced TNF-α secretion. The data is presented as the mean ± SD of triplicate measurements in four field repeats.

**Table S1: PCA analysis of YGQ leaf extracts.**

| principal component | ETDW (%) | ET (%) | EA (%) | HE (%) |
| --- | --- | --- | --- | --- |
| PC1 | 20.2 | 25.2 | 21.6 | 15.2 |
| PC2 | 13.7 | 12.7 | 13.4 | 11.9 |
| PC3 | 9.9 | 10 | 11.9 | 8.2 |
| PC4 | 8.3 | 8.6 | 7.9 | 9.2 |
| PC5 | 7.5 | 6.7 | 6.5 | 7.5 |
